# Employees and the National Insurance Act

**Published:** 1912-03-30

**Authors:** 


### NATIONAL INSURANCE ACT DEPARTMENT.

### EMPLOYEES AND THE NATIONAL INSURANCE ACT.

### IV.—Benefits Receivable under the Act—(*continued*).

In our last issue we set out a list of the standard benefits provided by the Act, and dealt in detail with the first two—namely, medical and sanatorium benefits. The next two benefits in the list are sickness benefit and disablement benefit, and these can conveniently be taken together.

Sickness and Disablement Benefits.

Sickness benefit is defined by the Act as "periodical payments whilst rendered incapable by work by some specific disease or by bodily or mental disablement, of which notice has been given, commencing from the fourth day after being so rendered incapable of work, and continuing for a period not exceeding twenty-six weeks."

Disablement benefit is the name given to the periodical payments to which the insured person may be entitled in the case of the disease or disablement continuing after the sickness benefit ceases to be payable, for so long as the insured person remains incapable of work by reason of the disease or disablement.

Rates of Benefit.

The ordinary rates of sickness benefit are 10s. per week for men and 7s. 6d. per week for women. In each case the amounts are payable throughout the whole period of twenty-six weeks.

The ordinary rate of disablement benefit is 5s. per week both for men and women. These ordinary rates, both for sickness and disablement benefits, are reduced in certain circumstances referred to later on.

It is important to note that no contribution is required from an insured person whilst in receipt of either benefit, but as the benefits may subsequently be reduced if the insured person is in arrears with his contributions beyond certain limits, it would appear to be a matter for consideration by the insured person if it is not to his advantage to pay the contributions whilst in receipt of benefit. This will be dealt with more fully when considering the question of arrears.

Waiting Periods.

Certain waiting periods are prescribed which must elapse before these benefits come into operation. In the case of sickness it is stipulated that at least twenty-six weeks must elapse since his entry into insurance and at least twenty-six weekly payments must have been paid in respect of him, before an insured person is entitled to the benefit. Disablement benefit will not be receivable until at least 104 weeks have elapsed since entry into insurance and at least 104 weekly contributions have been paid. In the case of illness commencing before an insured person is qualified for benefit, the benefits will become payable as soon as he is so qualified.

For the purpose of calculating the rate of benefit any two periods of sickness, unless separated by an interval of at least twelve calendar months during which at least fifty weekly contributions have been paid, will be reckoned as one illness. Thus if an insured person is ill and receives the full sickness pay for a period of, say, twenty-four weeks before declaring off the fund, and then makes another claim within twelve months of so ceasing to receive the benefit, the renewed illness, which may be either a recurrence of the previous malady or an entirely different disease, will only entitle him to full sickness benefit for a further two weeks, after which, if the sickness continues, the disablement benefit will come into operation if he is entitled to it.

Incapacity to Work.

The primary qualification for the receipt of sickness benefit by an insured person is the incapacity to work. This incapacity must arise either by some specific disease or by bodily or mental disablement. In the Friendly Societies some such rule has been common, but in a number of societies the members have been allowed to undertake certain forms of light labour whilst in receipt of sickness allowances, the general conception being that incapacity to work means incapacity to follow the usual or regular employment. The rules of Friendly Societies almost invariably provide a number of regulations as to the conduct of the members whilst in receipt of sickness allowances, such, for instance, as the hours of the day during which the member may be absent from home, and as to visitation by his fellow members. Recognising the importance of these requirements as a check against malingering, it is laid down in the Act that the rules of all approved societies as to the conduct of members whilst in receipt of sickness benefit shall be in a form to be prescribed by the Insurance Commissioners.

Notification of Illness.

The next matter to observe is that notice of sickness must be given. In the case of a member of an approved society this notice will have to be sent to the secretary of such society, and in the case of a deposit contributor to the local insurance committee. The notice will have to be furnished on a form supplied for the purpose, and it will have to be accompanied by a medical certificate or other sufficient evidence of incapacity and of its cause. According to the specimen sets of rules published by the Insurance Commissioners it will be necessary for a rule to be adopted by every approved society by virtue of which the committee of management, or similar governing body, shall require a medical certificate of incapacity for work to be furnished each week, or at such longer intervals as may be determined, while the member is drawing either sickness or disablement benefit. Notice will also have to be given under the rules as soon as the insured person becomes capable of work, and such penalties as are provided by the Act may be inflicted by the rules of the society to which the member belongs if such rules are infringed.

### Residence Outside the United Kingdom.

Residence outside the United Kingdom does not necessarily preclude the insured person from the receipt of sickness and disablement benefits. For the general purposes of the Act the Isle of Man and the Channel Islands are not included within its scope, but the Act provides that sickness and disablement benefits must not be disallowed whilst an insured person is temporarily resident in either of the islands. When an insured person is temporarily resident outside the United Kingdom elsewhere than in the Isle of Man or in the Channel Islands the approved society of which he is a member, or the insurance committee in the case of a deposit contributor, may allow him to continue to receive sickness or disablement benefit. This last provision would seem to be designed to meet the case of an insured person, already in receipt of either of the benefits, who is ordered abroad on account of his health, but it must be noted that the extension of the benefit is at the option of the society or committee.

### Commencement of Sickness Benefit.

It must be particularly noted that sickness benefit is not payable for the first three days of sickness. The custom of Friendly Societies has been to pay sick benefit from the first day's illness, and the variation of the State scheme from this common practice raised considerable opposition on the part of the Friendly Societies. It was in conciliation of their demands that the benefit was extended from thirteen weeks, as provided in the Bill in its original form, to twenty-six weeks, as it now stands in the Act. A further concession obtained by the Friendly Societies in this respect was that any recurrence of claim for sickness benefit within a year of its former receipt should date not from the fourth day of such recurrence but from the first day.

The estimated cost of the extension of the benefit to cover the first three days of sickness was £600,000 per annum, and the House decided that this money could be used to the greater advantage of the insured by providing for sickness of a prolonged nature, rather than in the mitigation of claims of very short duration. The right to sickness and disablement benefits ceases on the attainment of age seventy, at which age, it will be remembered, the contributions cease to be payable.

### Reductions of Benefits.

In certain cases the rates of benefit will be reduced either on account of the age of the insured member, or of special conditions affecting his employment, or by reason of the fact that his contributions are in arrear. We shall deal with these modifications of the ordinary benefits when considering the application of the Act to special classes of insured persons.

